# Willow Leaves' Extracts Contain Anti-Tumor Agents Effective against Three Cell Types

**DOI:** 10.1371/journal.pone.0000178

**Published:** 2007-01-31

**Authors:** Hany A. El-Shemy, Ahmed M. Aboul-Enein, Khalid Mostafa Aboul-Enein, Kounosuke Fujita

**Affiliations:** 1 Department of Biochemistry, Faculty of Agriculture, Cairo University, Giza, Egypt; 2 Department of Clinical Pathology, National Cancer Institute, Cairo University, Cairo, Egypt; 3 Graduate School of Biosphere Sciences, Hiroshima University, Hiroshima, Japan; The Scripps Research Institute, United States of America

## Abstract

Many higher plants contain novel metabolites with antimicrobial, antifungal and antiviral properties. However, in the developed world almost all clinically used chemotherapeutics have been produced by in vitro chemical synthesis. Exceptions, like taxol and vincristine, were structurally complex metabolites that were difficult to synthesize in vitro. Many non-natural, synthetic drugs cause severe side effects that were not acceptable except as treatments of last resort for terminal diseases such as cancer. The metabolites discovered in medicinal plants may avoid the side effect of synthetic drugs, because they must accumulate within living cells. The aim here was to test an aqueous extract from the young developing leaves of willow (*Salix safsaf*, Salicaceae) trees for activity against human carcinoma cells in vivo and in vitro. In vivo Ehrlich Ascites Carcinoma Cells (EACC) were injected into the intraperitoneal cavity of mice. The willow extract was fed via stomach tube. The (EACC) derived tumor growth was reduced by the willow extract and death was delayed (for 35 days). In vitro the willow extract could kill the majority (75%–80%) of abnormal cells among primary cells harvested from seven patients with acute lymphoblastic leukemia (ALL) and 13 with AML (acute myeloid leukemia). DNA fragmentation patterns within treated cells inferred targeted cell death by apoptosis had occurred. The metabolites within the willow extract may act as tumor inhibitors that promote apoptosis, cause DNA damage, and affect cell membranes and/or denature proteins.

## Introduction

Since human civilizations began, people have used plants for medicine [1]. In fact, until this century, plant derived treatments, faith, and surgery were the only treatments available. Among developed countries people seeking alternative medicines to avoid the side effects and expenses of synthetic medications have turned back to herbs to treat a wide array of ailments [1].

Traditional medicine has a long history of serving peoples all over the world [2]. Medicinal plants were an important element of indigenous medical systems that has persisted in developing countries. The enthnobotanic tradition and ubiquity of plants provides a rich resource for natural drug research and development. The plant kingdom was estimated to produce over 500,000 natural products and about 40–80 thousand per plant species [3]. Epidemiological and animal studies have demonstrated that plant-derived dietary constituents of food play an important role in the prevention of disease [4]. A number of food components have been identified that inhibit the initiation and progression of cancer or otherwise influence the potential for disease outcome [5]. For example, some epidemiological studies showed a close association between low incidence of coronary heart disease and breast cancer [6] and moderate consumption of red wine containing natural polyphenolic compounds [7].

Recently, the use of traditional medicine based on plants has received considerable interest [8]. There are national and indigenous rights over plant derived resources. Basic scientific investigations based on medicinal plants and indigenous medical systems have increased. A screening program was initiated by Leven et al. [9] that identified many antibacterial antifungal, antiviral, antiparasitic, and other pharmacologically active substance activities in higher plants [10].

Fiehn et al. [11] used Mass Spectrometry to identify and measure the abundance of hundreds of compounds in extracts from several plant species including *Arabidopsis thaliana*, and potato (*Solanum tuberosa*). More recently metabolite profiles that encompass thousands of metabolites have been developed using FT-ICR-MS in tobacco [12].

The genus Salix contained about 300 species. Salix has been a used in many traditional treatments [13] but is famous as the source of salicylic acid (Aspirin) that is stored in abundance just under the tree bark [14]. Aspirin is now synthesized in vitro and is free of impurities and co-metabolites. It has been used to reduce blood pressure, to thin blood by reducing the platelet count and to treat headaches. About 32 novel compounds have been identified in Salix sp. though no exhaustive metabolite profiles were available in 2006. In previous studies the active principles of the willow leaf extracts (Salicin and Saliginin chemicals related to salicylic acid) were shown to be anti-leukemia agents [13]. This work investigated the effect of Salicin and Saliginin in willow leaf extracts on the viability of tumor cells.

## Materials and Methods

### Samples Preparation

The willow leaves (*Salix safsaf* L.) were collected from the Salix farm of the Faculty of Agriculture, Cairo University, Giza, Egypt. On the day of harvest the young leaves (newly emerged) were extracted in hot water (at 10% w/v). About 10 g of fresh leaves were boiled (at 100°C) in 100 ml distilled water for 20 min, then filtered through a sterilized Miracloth and centrifuged at 15,000 g for 15 min. The Salicin concentration was about 750 ng/ml. The organic solvent extraction was carried out with the plant leaves as follows; 80 g leaves were extracted consecutively at room temperature with petroleum ether (at 40–60°C), that was followed by diethyl ether, chloroform, acetone, and finally with 70% (v/v) ethanol. The solvent of each extract was removed by distillation at a low temperature.

The study was performed on cells harvested from adult leukemia patients or healthy relatives, aged 18–65 years that were admitted to the National Cancer Institute, Cairo University. International protocols governing the ethical treatment of patient were followed. In addition animals were transplanted with EACC from an immortal culture obtained from National Cancer Institute, Cairo University, and maintained at mice transplanted line. International protocols governing the ethical treatment of animals were followed.

### Viability of Tumor Cells

The experimental samples were taken from healthy volunteers (6 samples) and leukemia patients that included 7 ALL (acute lymphoblastic leukemia) and 13 AML (acute myeloid leukemia, immature monocytes) patients. ALL and AML had been diagnosed by peripheral blood and bone marrow examination, cytochemistry (and immunological markers in some cases). Mononuclear cells were separated from other blood cells by Ficoll hypaque density gradient (Pharmacia, Uppsala, Sweden). The cells were then washed with three changes of PBS. The cell counts were adjusted to 10^5^ cells/0.1 ml (counting both mature and immature cells). The culture medium was prepared using modified Earle's salt with 1.2 g/l sodium carbonate and L-glutamine (Gibco, Grand island, USA), 10% inactivated fetal bovine serum (Gibco), and 100 units ml^−1^ penicillin and 100 µg ml^−1^ streptomycin.was added. The medium was filtered through 0.22 µm Millipore filter, one ml of which was transferred into a 1.8-ml screw-capped sterile plastic tube. Next, 0.1 ml of the cell suspension containing 10^5^ cells was added to each of 5 tubes. To three of the tubes, 0.1 ml of the willow extract was added, while the other two tubes served as negative and positive controls. Culture medium was used instead of the willow extract for the negative control and the willow extract was added to the cells from healthy volunteers as a positive control. The tubes were incubated at 37°C in the presence of 5% (v/v) CO_2_ for 24 h (dark condition, humidified air). The cells were tested for their viability using the trypan blue exclusion test [15]. Two hundred cells were counted, and the percentage of viable cells was estimated.

Cells of EACC were obtained from transplanted animals and then washed with three changes of PBS. The cell counts were adjusted to 10^5^ cells/0.1 ml. The purification methods and treatment protocols described above were used to determine the effects of willow extracts on the viability of EACC.

### Tumor Transplanted Animals

A total of 36 normal female Swiss albino mice weighing between 20–25 g each and fed a normal diet were used as follows: One group of mice were spared a cell transplant and referred to as the control (negative). The second group was transplanted to the intraperitoneal (i.p) cavity with EACC at 2×10^6^ cells (0.2 ml) (positive control). The third group was transplanted with EACC like the second group and each mouse was daily forced to ingest orally via stomach tube about 0.2 ml of an aqueous willow extract (10% w/v) in addition to the normal diet. The fourth group were treated daily like the third group except with 0.6 ml willow extract.

### DNA Extraction

DNA was extracted from mature (normal cells) and immature white blood cells (leukemic cells) before and after treatment with willow extract. Cells were washed with PBS and then lysed in cold lysis solution (5 mmol/L of Tris, pH 7.4, 20 mmol/L of EDTA, 0.5% Triton X-100) for 20 min [16]. Cell lysates were centrifuged at 27,000 *g* for 15 min, and DNA was extracted from the aqueous phase with phenol:chloroform:isoamyl alcohol (25∶24∶1,v/v/v) containing 0.1% (w/v) hydroxyquinoline. DNA was precipitated with 0.3 mol/L of sodium acetate and 2 volumes of cold 100% (v/v) ethanol. Agarose gel electrophoresis (1% w/v) at 30 mA for 2 h followed by UV fluorescence was used to determine the degree of apoptotic DNA fragmentation [16].

## Results and Discussion

**Figure 1:**
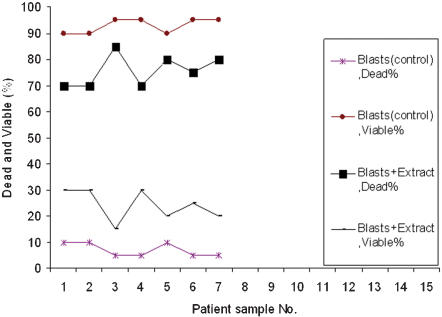
The effect of willow young leaves extracts on the viability of ALL cells. 7 ALL patients (acute lymphoblastic leukemia) 0.1 ml of the cell suspension containing 10^5^ cells was added to 5 tubes. To three of the tubes, 0.1 ml of the willow extract was added, while the other two tubes served as negative and positive controls. Culture medium was used instead of the willow extract for the negative control and the willow extract was added to the cells from healthy volunteers as a positive control. The tubes were incubated for 24 h and tested for their viability using the trypan blue.

**Figure 2:**
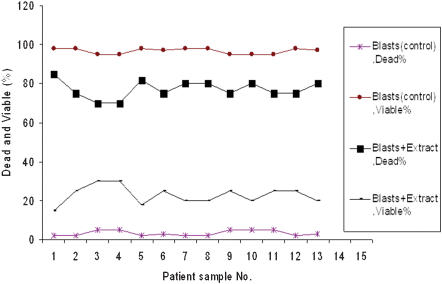
The effect of willow young leaves extracts on the viability of AML cells. 13 AML patients (acute myeloid leukemia) 0.1 ml of the cell suspension containing 10^5^ cells was added to 5 tubes. To three of the tubes, 0.1 ml of the willow extract was added, while the other two tubes served as negative and positive controls. Culture medium was used instead of the willow extract for the negative control and the willow extract was added to the cells from healthy volunteers as a positive control. The tubes were incubated at tested for their viability using the trypan blue.

After 24 h incubation of the mononuclear ALL cells with salix extract, a remarkable destruction of lymphoblasts (75%) occurred that was significantly higher than the control (7%; Figure 1). In the case of mononuclear AML cells similar trends were observed, the mean viability of willow extract treated cells was 26.2% when compared to the control (96.9%; Figure 2). The willow extract was incubated with normal mononuclear cells as a positive control from healthy volunteers (6 samples). The results showed that there was no significant difference in killing the cells (mean 7.8% and 6.7%) when compared to 3.1% in the control (negative control). From these data, it is clear that leukemia cells were more vulnerable to the extract.

The anti-tumor effects of willow leaf extracts were novel. However, willow leaves have long been used in folk medicine as an antirheumatic, analgesic, and antipyretic herbal medicine. In connection with antileukemia, the allamandin derivatives that are extracted with water and/or ethanol from *Allamanda catharica* (Apocynaceae) showed significant activity in vivo against the p-388 leukemia in the mouse [17].

Various fractions including different leaf extracts (*Salix safsaf*) by successive solvent extractions were tested for their antileukemic activity on acute myeloid leukemia (AML) cells and acute lymphoblastic leukemia (ALL). The fractions of each crude extract were dissolved in a saline solution after removing the solvent and incubated with the leukemia cells. The results showed that a fraction of the willow leaves extracted with nonpolar organic solvents (petroleum ether, ether, and chloroform) had a very low destructive effect on tumor cells as shown by a higher viability 80–90%. Destruction of each extract ranged between 1.5–2.9%. of each extract. However, a major destructive effect on AML, ALL and carcinoma (EACC) cells was obtained by a fraction of the polar organic solvents (water and 70% ethanol).

**Figure 3:**
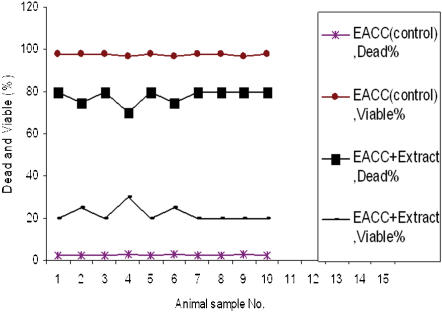
The effect of willow young leaves extracts on the viability of Ehrlich asites carcinoma cells (EACC). 0.1 ml of the cell suspension containing 10^5^ cells was added to 4 tubes. To three of the tubes, 0.1 ml of the willow extract was added, while the other tube served as negative control. Culture medium was used instead of the willow extract for the negative control. The tubes were incubated for 24 h and tested for their viability using the trypan blue.

The viability of tumor cells (EACC) after incubation with water extract of salix leaves were greatly affected (80% cell death) compared to untreated cells (2% cell death) (Figure 3). From this observation, it is clear that the antitumor activity of the willow leaves was mostly due to compounds that were soluble in water and/or ethanol. These active ingredients were easily dissolved in hot water, and in turn could be used as a natural antitumor medicine. The phenolic compounds, most glycosides, and many types of tannin will dissolve in water or ethanol solutions [18]. Therefore, these groups of compounds may contain the major active components for the destruction of leukemia and carcinoma cells. Salicin was the primary compound in salix leaves that could be dissolved in water and ethanol. Salicin was identified by comparing the retention time and spectral characteristics of the extract to those of the reference compounds by GCMS and HPLC. Here, the salicin standard (Sigma, St. Louis, USA) was tested for its antileukemic and anti-carcinoma cells effect. Salicin (0.1 ml of a 0.75 mg/ml) was added to the suspension of 10^5^ AML, ALL and EACC cells. The results showed that the destruction of myeloblasts and tumor cells (70–75%) was significantly higher than those in the control specimens. From these observations, salicin may be the major component that shows the anti-tumor effect but other metabolite may increase the potency of the willow extract compared to pure salicin.

**Figure 4:**
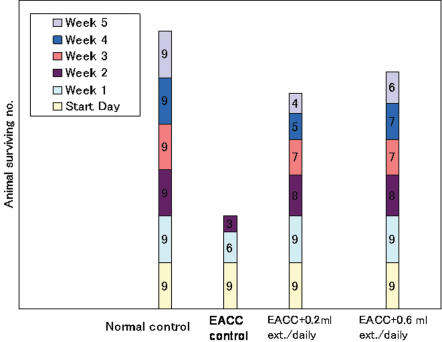
Effect of salix water extract on surviving of transplanted animals (EACC). One group fed on normal diet (negative control), second group was fed on normal diet and transplanted to the intraperitoneal (i.p) cavity with EACC at 2×10^6^ cells (0.2 ml) (positive control), third group fed the normal diet and EACC infected then each mouse daily forced to ingest orally via stomach tube about 0.2 ml of an aqueous willow extract (10% w/v), fourth group were treated daily like the third group except with 0.6 ml willow extract.

Transport of salicin and saligenin into erythrocytes was rapid for saligenin (1 min to saturation) compared with salicin (4 h to saturation)[19]. The process was reversible, exhibiting a rapid release for saligenin and slower release for salicin [19]. Both saligenin and salicin bind to human serum albumin, but the former has the significantly higher affinity [19]. Ehrlich asites tumor (EACC) in mice had been used as a model for rapidly growing tumors where various experimental anticancer agents can be applied. Herein analyses of transplanted animals before and after tumor transplantation were correlated with willow extract administration. The results showed that water extract of willow leaves delayed the death and inhibited the tumor growth of transplanted animals compared with untreated transplanted animals that died within two weeks (Figure 4). However, the active principles in water extracts modified the features of EACC hampering their growth after transplantation.

**Figure 5:**
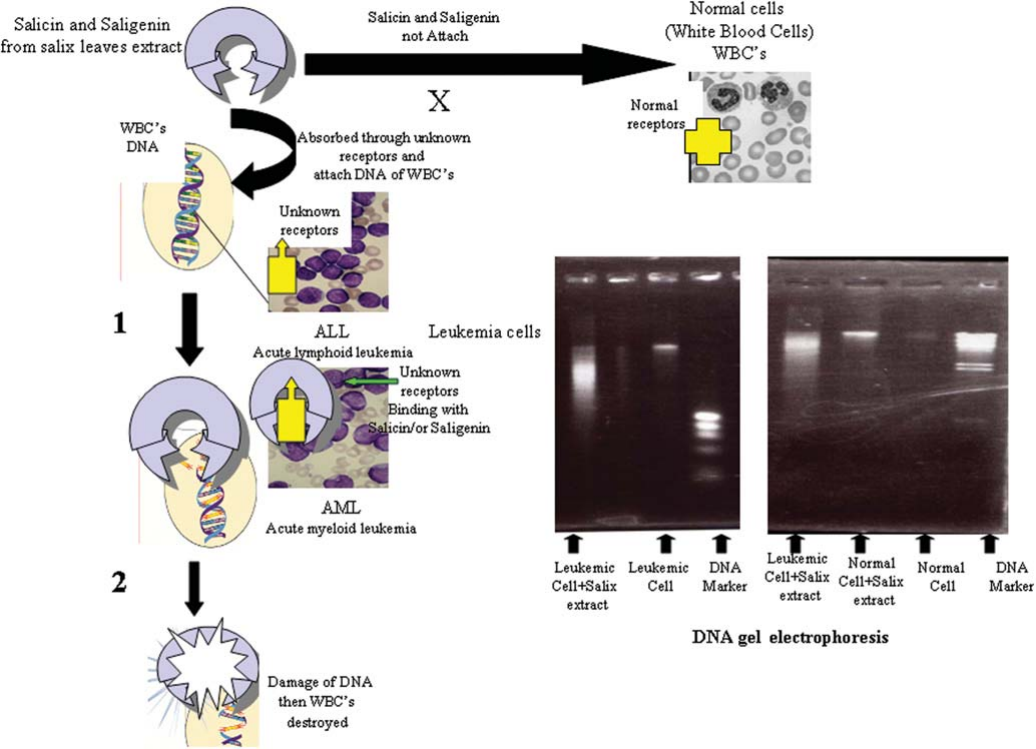
The mode of action of the Salicin and Saligenin to destroy immature White Blood Cells (WBC's) in leukemia patients.

Side effect tests on animals showed salix extract did not induce widespread cell damage. There was no difference between the feeding and non-feeding animals in blood glucose, GOT, GPT and alkaline phosphatase activity. Salicin was partially metabolized to saligenin and salicylic acid (a known effective compound) after incubation with homogenized kidneys from rats [20]. Saligenin was transformed to salicylic acid by homogenized liver, lung, and kidney extracts. Gentisic acid was qualitatively detectable in the homogenized liver after incubation with saligenin [20]. The transport of salicin and saligenin through the isolated intestinal wall was confirmed using the closed-off posterior section of the male rat intestine. When salicin and saligenin were injected into the closed intestine, both passed unchanged through the ileal wall. Saligenin appeared to penetrate the intestinal wall faster than salicin [21]. The metabolites were equivalent to more than 86% of the administered salicin, that were recovered in 24-h urine: salicyluric acid (51%), salicyl glucuronide (14%), salicylic acid (12%), gentisic acid (5%), and saligenin (49%) together with a small amount of unchanged salicin [22]. Based on these findings, it is speculated that the tumor cells may emit some signaling substances for salicin and saligenin receptors. The willow compounds may bind with receptors on the surface of tumor cells and penetrate into the cells. The cells could be killed through denaturation of some enzymes and proteins that are induced by salicin and saligenin (Figure. 5). Signaling between cells is commonly regarded as the most important mechanism by which cell-type differences arise in development and by which patterns of tissue organization are established [23]. At almost every stage in development, cells emit and receive signals from other nearby cells, and these signals are necessary for normal differentiation and function [23].

Little is known about the bioavailability, absorption and metabolism of secondary metabolites of salix tree in humans and it is likely that different groups of compounds have different pharmacokinetic properties. The study reported here describes a simple mode of action of salicin and saligenin compounds to bind with the immature white blood cells only and destroy them with apoptosis associated DNA damage (Figure 5). Highly DNA damaged in leukemic cells incubated with salix extract was surprising when normal cells incubated with salix extract remained unaffected (Figure 5).

Even assuming unknown receptors in the surface of leukemic cells may be binding with salix extract compounds and leading to DNA destruction (Figure 5) the mode of action of salicin and saligenin is not clear. The compounds from salix extract shown in Figure 5, will need more clinical experiments to elucidate the receptors and transduction pathways induced in leukemic cells.

Gao and his coworker [16] have investigated the resveratrol-induced DNA fragmentation in 32Dp210 leukemic cells. Resveratrol induced apoptosis in 32Dp210 cells as supported by the induction of internucleosomal DNA fragmentation and the cleavage of procaspase-3 in resveratrol treated cells [16]. These results supported the previously reported apoptosis-inducing activity of resveratrol against tumor cell lines [24.25].

In conclusion the message from Arabian and Middle East researchers to medical scientists over the world was to get back in nature and search for new drugs, to avoid the side effect of chemical therapy and to help patients recover from acute diseases.
